# New Recombinant Mycobacterium bovis BCG Expression Vectors: Improving Genetic Control over Mycobacterial Promoters

**DOI:** 10.1128/AEM.03677-15

**Published:** 2016-04-04

**Authors:** Alex I. Kanno, Cibelly Goulart, Henrique K. Rofatto, Sergio C. Oliveira, Luciana C. C. Leite, Johnjoe McFadden

**Affiliations:** a Centro de Biotecnologia, Instituto Butantan, São Paulo, SP, Brazil; b Programa de Pós Graduação Interunidades em Biotecnologia USP-IPT-IB, São Paulo, Brazil; c Departamento de Bioquímica e Imunologia, Universidade Federal de Minas Gerais, Belo Horizonte, MG, Brazil; d Department of Microbial and Cellular Sciences, Faculty of Health and Medical Sciences, University of Surrey, Guildford, Surrey, United Kingdom; University of Buenos Aires

## Abstract

The expression of many antigens, stimulatory molecules, or even metabolic pathways in mycobacteria such as Mycobacterium bovis BCG or M. smegmatis was made possible through the development of shuttle vectors, and several recombinant vaccines have been constructed. However, gene expression in any of these systems relied mostly on the selection of natural promoters expected to provide the required level of expression by trial and error. To establish a systematic selection of promoters with a range of strengths, we generated a library of mutagenized promoters through error-prone PCR of the strong P_L5_ promoter, originally from mycobacteriophage L5. These promoters were cloned upstream of the enhanced green fluorescent protein reporter gene, and recombinant M. smegmatis bacteria exhibiting a wide range of fluorescence levels were identified. A set of promoters was selected and identified as having high (pJK-F8), intermediate (pJK-B7, pJK-E6, pJK-D6), or low (pJK-C1) promoter strengths in both M. smegmatis and M. bovis BCG. The sequencing of the promoter region demonstrated that it was extensively modified (6 to 11%) in all of the plasmids selected. To test the functionality of the system, two different expression vectors were demonstrated to allow corresponding expression levels of the Schistosoma mansoni antigen Sm29 in BCG. The approach used here can be used to adjust expression levels for synthetic and/or systems biology studies or for vaccine development to maximize the immune response.

## INTRODUCTION

Mycobacterium bovis BCG is currently the world's most widely used vaccine and has been given to more than three billion people, making it a very attractive prospect for the development of a live recombinant BCG (rBCG) multivaccine (1). The first generation of rBCG vaccines was developed in the 1990s as rBCG strains that expressed homologous and heterologous antigens from a wide range of pathogens (2–5). Stability of the heterologous (or native) gene(s) in BCG was usually obtained by cloning it on a plasmid or chromosomally integrative vector with expression achieved by placing it under the control of a range of mycobacterial promoters (2). The heat shock protein promoters (P_Hsp60_ from BCG and P_Hsp70_ from M. tuberculosis), the IS*900* open reading frame promoter (P_AN_ from M. paratuberculosis), and the mutated β-lactamase promoter (P_BlaF*_ from M. fortuitum) are the ones used most, as reviewed in reference 2. However, the lack of knowledge of the transcriptional mechanisms regulating mycobacterial promoters results in unpredictable gene expression levels. Yet the level of antigen expression is likely to be a crucial factor in both the strength and the pattern of subsequent immune responses. For example, a study in which expression of M. tuberculosis antigen 85B (Ag85B) in rBCG was placed under the control of a limited set of promoters found that increasing promoter activity caused a skewing of the immune response to Ag85B in mice from a mixed Th1/Th2 response to a predominantly Th1 response (6). In another study, increasing expression of M. tuberculosis 19-kDa lipoprotein led to complete abrogation of the protective efficacy of BCG by polarizing the host immune responses to the Th2 subtype (7). Another important factor for any vaccine vector is stability and the associated metabolic burden, which can lead to loss of antigen expression and/or premature elimination of the vector in the host because of loss of fitness (8). Indeed, loss of vector during replication in the host is thought to have been responsible for the failure of an rBCG vaccine expressing outer surface protein A (OspA) of Borrelia burgdorferi to generate an appropriate immune response in a clinical trial, despite the apparent efficacy of the vaccine in mice (9). Our own earlier studies similarly demonstrated vector instability and a lack of correlation of immune responses in mice with expression levels *in vitro* (10).

To improve the genetic control of gene expression in mycobacteria, different tools and strategies can be used, such as inducible promoters (11), synthetic genes with codon-optimized sequences (12, 13), substitution of the initiation codon (14), addition of Shine-Dalgarno sequences (15), an increase in the plasmid copy number (16), or integration into the mycobacterial genome (17). However, once again, expression levels are often unpredictable and may be further complicated by regulatory influences on expression levels, particularly during growth in the host environment. In this work, as a first step toward a more rational approach to vaccine vector design, we engineered the mycobacteriophage promoter P_L5_ (18). Our aim was to generate a set of mycobacterial promoters that can be used to obtain a predictable range of gene expression levels in both M. smegmatis and M. bovis BCG.

## MATERIALS AND METHODS

### Ethics statement.

This study was conducted in accordance with the recommendations and with the approval of the Committee on the Ethics of Animal Experiments of the Butantan Institute under protocol 594/09.

### Bacterial strains and plasmids.

**FIG 1:**
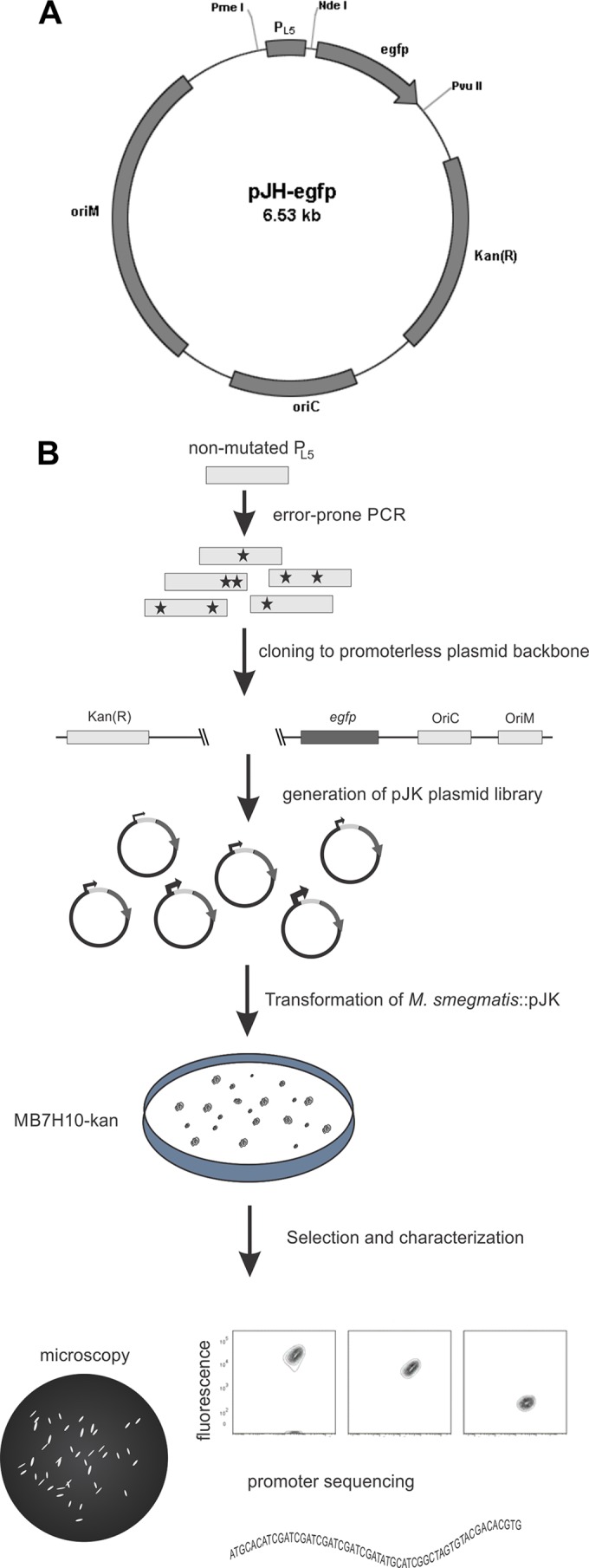
Schematic representation of pJH-*egfp* and the methodology used to generate a functional promoter library. (A) pJH-*egfp* contains the P_L5_ promoter upstream of the *egfp* sequence, a kanamycin resistance marker [Kan(R)], and origins of replication in E. coli (oriC) and mycobacteria (oriM). (B) The P_L5_ promoter was submitted to error-prone PCR and used to drive the expression of *egfp. M. smegmatis* transformants were selected by kanamycin resistance, and functional promoters were screened within fluorescent colonies. Selected colonies spanning a wide range of fluorescence levels were further analyzed by microscopy, specific promoter mutations, and single-cell fluorescence by flow cytometry. (Adapted from reference 26.)

Escherichia coli DH5α (19) (Invitrogen) was used in all cloning steps with lysogeny broth (LB) or LB plates with kanamycin (20 μg/ml) for selection of transformants. M. smegmatis strain MC^2^155 (20) and M. bovis BCG strain Pasteur (21) were grown at 37°C in Difco Middlebrook 7H9 broth (Becton, Dickinson) enriched with 10% (vol/vol) oleic acid-albumin-dextrose-catalase (OADC), 0.5% glycerol, and 0.05% Tween 80 (MB7H9) containing kanamycin when necessary. Electrocompetent M. smegmatis and BCG were transformed by electroporation as previously described (22), and transformants were selected on Middlebrook 7H10 agar plates with OADC (MB7H10) and kanamycin. All primers were purchased from Eurofins MWG Operon (Ebersberg, Germany), and all restriction enzymes were from New England BioLabs (Hitchin, United Kingdom). GoTaq DNA polymerase (Promega, Southampton, United Kingdom) was used in all PCRs. The expression cassette of the pBRL8 plasmid (kindly provided by William Jacobs, Jr., Yeshiva University, New York, NY) containing the P_L5_ promoter and the *egfp* gene was PCR amplified with primers 5′-TAGGGTACC**TCTAGA**GGAAACAGCTATGACCAT-3′ (the KpnI and XbaI restriction sites are underlined and in bold, respectively) and 5′-TAGATCGAT**CAGCTG**TTACTTGTACAGCTCGT-3′ (the ClaI and PvuII restriction sites are underlined and in bold, respectively). This fragment was cloned into pJH152 (kindly provided by Stewart T. Cole, Ecolé Polytechnique Fédérale de Lausanne, Lausanne, Switzerland) between the KpnI and PvuII restriction sites, thus generating pJH-*egfp* (Fig. 1A). Next, error-prone PCR of the promoter sequence was carried out in the presence of 25 μM 8-oxo-dGTP and 5 μM dPTP (Jena Bioscience, Germany), along with 400 μM natural deoxynucleoside triphosphates (dNTPs) (Fig. 1B). According to the manufacturer's instructions, 20 rounds of amplification were performed with primers 5′-TAG**GTTTAAAC**AAACGGAAACAGCTATGACCAT-3′ (the PmeI restriction site is in bold) and 5′-TAG**CATATG**CGATCTCCCTTTCCCGT-3′ (the NdeI restriction site is in bold). Plasmid pJH-*egfp* and the PCR product were digested with PmeI and NdeI, resolved in an agarose gel electrophoresis to remove the original cassette and clean up the PCR product, and further purified with the QIAquick PCR Purification kit (Qiagen, Hilden, Germany). Before ligation, the digested vector was treated with calf intestinal alkaline phosphatase (Promega) in accordance with the manufacturer's instructions for the dephosphorylation of 5′ overhangs and purified once again. Digested fragments were allowed to ligate with T4 DNA ligase (Promega) at 16°C for 16 h and used to transform E. coli DH5α. Plasmid DNA was extracted from approximately 2,000 colonies in a pool, generating a pJK plasmid library that, in turn, was used in the electroporation of M. smegmatis MC^2^155 with a Bio-Rad Gene Pulser II apparatus (Bio-Rad, Hercules, CA). Transformants were allowed to grow for 3 to 5 days on MB7H10-kanamycin plates (Fig. 1B). Plasmid pJH137 was digested with restriction enzymes XbaI and NdeI to remove the heat shock promoter P_Hsp60_ and insert it in place of P_L5_ of pJH-*egfp*, generating pJHsp60. Plasmid pET-21 containing the Sm29 sequence to produce rSm29 protein was previously constructed (23).

### Screening of fluorescent M. smegmatis.

After visualization of M. smegmatis transformants by eye, approximately 200 colonies were isolated and grown in MB7H9-kanamycin. Samples were allowed to grow for 16 h and screened on 96-well black plates in duplicate with a PerkinElmer Victor 3 fluorescence multiplate reader (PerkinElmer, Waltham, MA) set up for excitation and emission wavelengths of 485 and 535 nm, respectively. Also, the optical density at 620 nm (OD_620_) of each sample was measured. The fluorescence of the strains was initially screened in fluorescence units (FU), and then, following normalization by OD, five strains were selected on the basis of measurements of relative FU (RFU). The plasmid in each of these transformants was extracted with the illustra plasmidPrep Mini Spin kit (GE Healthcare Life Sciences, Freiburg, Germany), and P_L5_ promoters were sequenced with the primer 5′-TCAGCTTGCCGTAGGTGGCA-3′.

### Characterization of GFP expression and *in vitro* growth of selected mutants.

Shaking flasks with MB7H9-kanamycin were inoculated with selected M. smegmatis/pJK in triplicate to an OD of ∼0.1. Culture turbidity and fluorescence were monitored every hour. For comparison, wild-type M. smegmatis strain MC^2^155 grown in MB7H9 was also analyzed. A FACSCanto II flow cytometer (Becton, Dickinson) was used to determine the single-cell fluorescence of gated bacterial cells on the basis of the median of fluorescein isothiocyanate (FITC; with 488-nm excitation and 500- to 560-nm emission filters). Turbidity was measured with an Ultrospec 2000 spectrophotometer (Pharmacia Biotech, Sweden). To compare the newly generated promoters with a widely used one, P_Hsp60_ was inserted in place of P_L5_. M. bovis BCG bacteria containing these plasmids were analyzed and compared with M. smegmatis constructs. Late-log-phase cultures of recombinant M. smegmatis strains were disrupted on ice with a GE 100 Ultrasonic Processor at half-maximum constant output, and the protein concentration in culture lysates was determined by the DC Protein Assay (Bio-Rad) with bovine serum albumin as the standard. Approximately 10 μg of soluble protein extracts was separated by SDS-PAGE and stained with Coomassie brilliant blue (Bio-Rad). The predicted molecular mass of GFP is 28.3 kDa (http://web.expasy.org/compute_pi/). Following destaining, the band corresponding to GFP's molecular weight was analyzed by densitometry with ImageJ software.

### Microscopy analysis of selected strains.

M. smegmatis/pJK strains were observed under a microscope. Late-log-phase cultures were washed twice with phosphate-buffered saline and placed on microscope slides. Images were acquired on an LSM 510 META confocal system (Zeiss, Germany) with a 488-nm laser for excitation and a 500- to 550-nm bandpass filter for emission.

### Fine-tuned expression of foreign antigen.

To assess the usefulness of the pJK library regardless of the downstream gene under its regulation, we used three plasmids containing the mutagenized promoters, pJK-F8, pJK-B7, and pJK-C1. For this model, we removed the *egfp* gene by digestion with NdeI and PvuII and cloned the partial sequence of the Schistosoma mansoni gene *sm29* (GenBank accession no. AF029222) codon optimized for expression in mycobacteria, as this antigen is currently considered a vaccine candidate (23), creating pJK-F8.*sm29*, pJK-B7.*sm29*, and pJK-C1.*sm29*. Codon optimization was performed by the DNA 2.0 company according to their patented algorithm. Codon-optimized *sm29* had an increased GC content (from 37 to 51%). BCG was transformed with these constructs and allowed to grow as described previously. Kanamycin-resistant clones were grown until late log phase, disrupted on ice, and quantified as previously described. Approximately 25 μg of total protein extracts was separated by SDS-PAGE, and proteins were electrotransferred to a polyvinylidene difluoride membrane (GE Healthcare) and blocked for 16 h at 4°C in a 5% nonfat dry milk solution. Western blotting of Sm29 expression used polyclonal mouse anti-rSm29 antibody (1:1,000) and a horseradish peroxidase (HRP)-conjugated anti-mouse antibody adsorbed to human serum proteins (KPL, Gaithersburg, MD) at 1:3,000. The chemiluminescent signal was detected with the Immobilon Western Chemiluminescent HRP Substrate (Merck, Darmstadt, Germany), and images were acquired with the LAS4000 digital imaging system (GE).

### Recombinant Sm29 protein and anti-rSm29 antibody production.

Recombinant Sm29 was expressed in E. coli BL21(DE3) (Invitrogen) transformed with pET-21 containing the Sm29 cDNA sequence and purified by metal affinity chromatography as previously described (24). To generate the primary antibody, three BALB/c mice were immunized subcutaneously with three doses of rSm29 (25 μg) adsorbed to aluminum hydroxide (250 μg) with 2-week intervals between doses. Fifteen days after the last dose, cardiac puncture of anesthetized mice was performed for blood collection and serum processing.

### Statistical analysis.

For statistical analysis, the GraphPad Prism 5 software (GraphPad, La Jolla, CA, USA) was used. The relationship between pJH-*egfp* and the pJK library in the initial screening was tested by two-tailed Spearman rank correlation. The fluorescence induced by pJK in M. smegmatis or BCG was analyzed by one-way analysis of variance (ANOVA) and Tukey's posttest. Correlation of interspecies fluorescence was tested with a nonparametric Spearman test. Growth curves were compared by nonlinear regression.

## RESULTS

### Generation of the P_L5_ promoter library.

**FIG 2:**
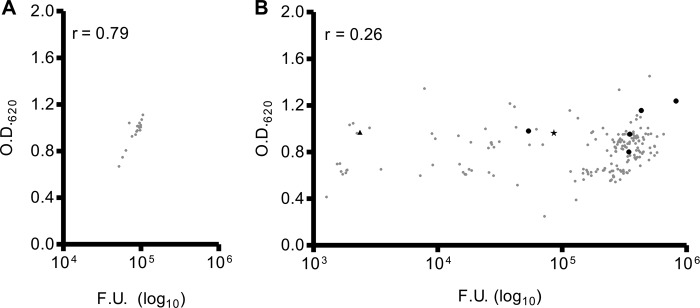
Screening of P_L5_ promoter mutant strength. (A) Clones of M. smegmatis/pJH-*egfp* with the nonmutated P_L5_ promoter show a correlation between growth (OD_620_) and fluorescence (log_10_ FU) (*r* = 0.79). (B) M. smegmatis transformed with a pJK plasmid library shows a variation in fluorescence independently of growth (*r* = 0.26). Wild-type M. smegmatis (no *egfp* gene) and M. smegmatis/pJH-*egfp* are represented by a black triangle and a star, respectively. The mutants selected for further analysis are represented by black circles. Statistical analysis was done by two-tailed Spearman rank correlation.

First, we used error-prone PCR (25) to generate a library of P_L5_ promoters and assess the strengths of variant sequences by cloning the promoters to drive enhanced green fluorescent protein (eGFP) expression (Fig. 1B). The wild-type P_L5_ promoter and the *egfp* gene were cloned into pJH152, generating pJH-*egfp* (Fig. 1A), which was used to transform M. smegmatis, thus representing the fluorescence induced by the original promoter, which correlated with bacterial growth (Fig. 2A). The pJK plasmid library was used to transform M. smegmatis, and the approximately 200 colonies named pJK-XA1 to -YH12 were screened by fluorimetry and demonstrated a wide variation in fluorescence levels, ranging between 10^3^ and 10^6^ FU (Fig. 2B).

### Characterization of selected M. smegmatis/pJK strains.

**FIG 3:**
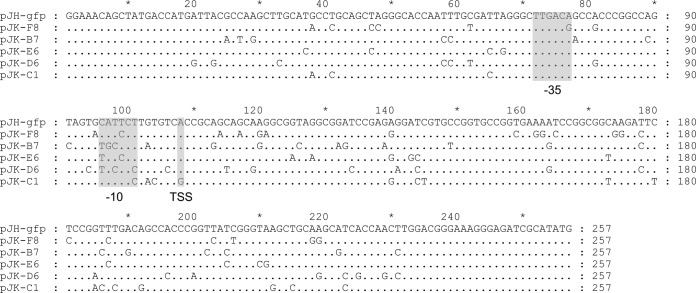
Sequence alignment of wild-type and mutated P_L5_ promoters in pJK plasmids. The promoter sequence containing the selected pJK plasmids was sequenced and compared to the original. Of 257 bp comprising the promoter region, 6 to 11% are altered in mutants; they also localized to the predicted −10 and −35 regions. pJK-C1 showed a mutation at the transcriptional starting site (TSS). The initiation codon is the last 3 bp in the sequence shown.

**FIG 4:**
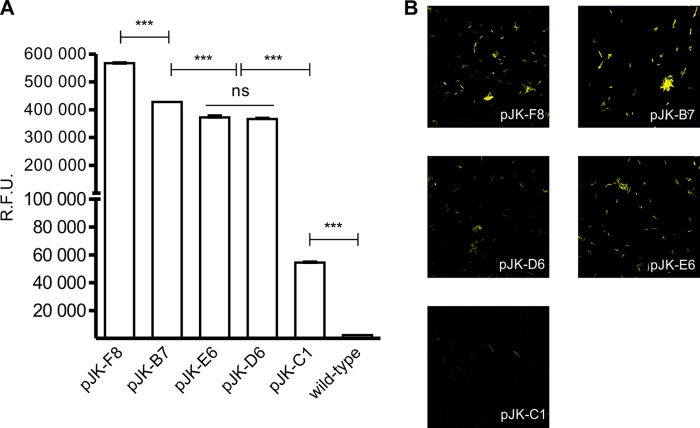
Error-prone PCR changes P_L5_ promoter strength. (A) Relative fluorescence (RFU) of selected transformants comprising the fluorescence range. (B) Microscopy of M. smegmatis/pJK demonstrating differential fluorescence with respect to promoter strength, with little variation among the bacillus population. Statistical analysis was done by one-way ANOVA (***, *P* < 0.001; ns, not significant).

On the basis of the initial screening, five strains covering the fluorescence range were selected for further analysis. First, the sequencing of selected promoters revealed multiple mutations with modification in 6 to 11% of their sequences (Fig. 3). Since the ODs of M. smegmatis/pJK library mutants varied between 0.3 and 1.5, the data were normalized and fluorescence levels were plotted in RFU (Fig. 4A). Likewise, confocal microscopy confirmed the differential expression of GFP where the fluorescence was distributed throughout the bacillus cell (Fig. 4B). To exclude the possibility that differential fluorescence would be a consequence of impaired growth or a distinct rate of GFP expression, selected M. smegmatis/pJK strains were evaluated for single-cell fluorescence during growth. Constant fluorescence was observed during the growth of each mutant strain (not shown). M. smegmatis/pJK-C1 was the only strain whose growth was demonstrated to be significantly different from that of the wild-type strain (see Fig. S1 in the supplemental material).

### Flow cytometry of M. smegmatis and M. bovis BCG/pJK strains.

**FIG 5:**
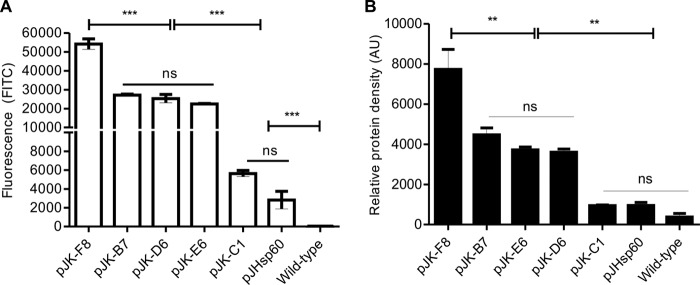
pJK plasmids induce differential expression in M. smegmatis. Late-log-phase cultures of M. smegmatis were transformed with selected pJK plasmids or pJHsp60. (A) Fluorescence of recombinant M. smegmatis harboring pJK plasmids analyzed by flow cytometry. (B) Relative density of the GFP band on SDS-PAGE analyzed by densitometry. Statistical analysis was done by one-way ANOVA (***, *P* < 0.001; **, *P* < 0.01; ns, not significant).

**FIG 6:**
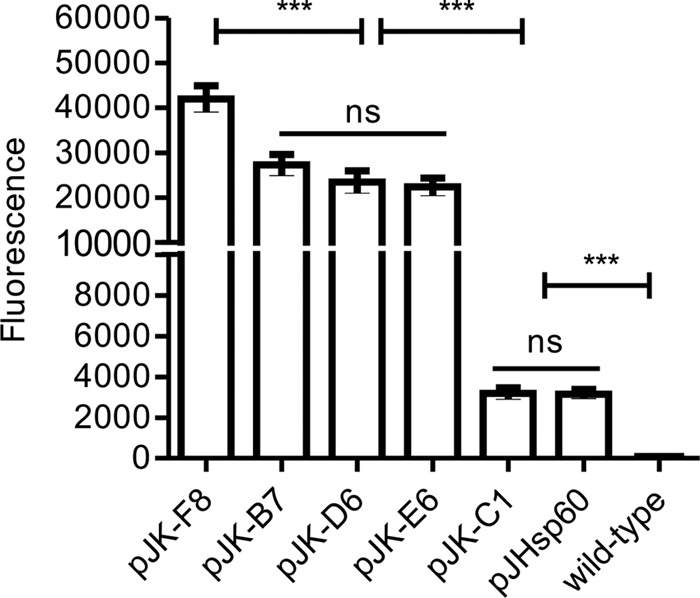
pJK plasmids induce differential fluorescence levels in M. bovis BCG. Late-log-phase cultures of BCG were transformed with pJK plasmids or pJHsp60, and fluorescence was analyzed by flow cytometry. Statistical analysis was done by one-way ANOVA (***, *P* < 0.001; ns, not significant).

The selected pJK plasmids were transformed into M. bovis BCG, and late-log-phase cultures were analyzed alongside recombinant M. smegmatis by flow cytometry. After gating of the bacilli's population, fluorescence was determined as the median FITC value. M. smegmatis transformed with pJK-F8 showed high fluorescence; M. smegmatis transformed with pJK-B7, pJK-E6, and pJK-D6 showed intermediate fluorescence; and M. smegmatis transformed with pJK-C1 showed strikingly low fluorescence (Fig. 5A). Recombinant M. smegmatis strains were also submitted to SDS-PAGE, and the bands corresponding to GFP were quantified by densitometry (Fig. 5B). The results demonstrated that the fluorescence intensity measured by flow cytometry correlated with the amount of protein produced (*r* value = 0.96) according to the strength of each promoter. BCG transformed with the same plasmids revealed a high correlation with the observed fluorescence of M. smegmatis (*r* value = 0.94) (not shown), where all of the mutants of both species maintained their high, intermediate, or low strength. pJHsp60 demonstrated a strength comparable to that of the lowest-expression vector, pJK-C1 (Fig. 6).

### Mutant promoters induce differential antigen expression.

**FIG 7:**
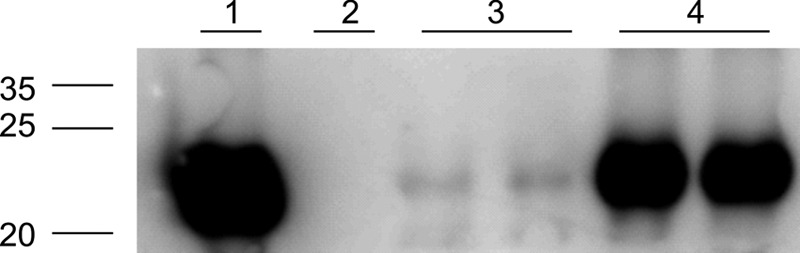
Western blot assay of rBCG strains differentially expressing Sm29 antigen through mutated P_L5_ promoters. Total protein extracts (25 μg) of wild-type BCG (lane 2), rBCG/pJK-C1.Sm29 (lanes 3), and pJK-B7.Sm29 (lanes 4) were used. Two independently isolated clones were analyzed by Western blot assay with anti-rSm29 antibodies. A 300-ng sample of rSm29 protein (lane 1) was used as a positive control. The values to the left are molecular sizes in kilodaltons.

To investigate the usefulness of these promoters for the expression of other heterologous genes, we replaced the *egfp* gene from pJK-F8, pJK-B7, and pJK-C1 with the codon-optimized *sm29* gene from S. mansoni. Since pJK-F8 produced no colonies after transformation, further work was done with pJK-B7 and pJK-C1. rBCG clones of these strains were grown to late log phase and lysed by sonication. Total protein extracts were analyzed by Western blot assay with specific anti-rSm29 antibodies. As predicted, we obtained a more intense immunoblot assay result with the rBCG/pJK-B7.Sm29 extracts (higher promoter activity) than with the reaction in the rBCG/pJK-C1.Sm29 extracts (lower promoter activity) at the same molecular weight as recombinant protein rSm29, but no immunoreaction was observed in wild-type BCG protein extracts (Fig. 7).

## DISCUSSION

Here we used the gene for eGFP as a reporter to measure the differential expression of this protein induced by mutations in the regulatory region of the mycobacteriophage L5 P_L5_ promoter sequence. The P_L5_ promoter regulatory sequence was subjected to random mutagenesis, and a library of promoters exhibiting different strengths, as measured by fluorescence, was selected. The gene for GFP is one of the reporters most often used to characterize promoter strength among many organisms in a quantitative fashion (26–28), including in mycobacterial species for promoter discovery, strength, and trafficking in the host (11, 29–32).

The observed 6 to 11% mutations in the regulatory sequence is consistent with the expected rate of 10 to 15% mutagenesis with equimolar concentrations of altered and natural dNTPs (25). A higher degree of mutation can span a wider range of downstream expression; however, it can also abolish expression by altering motifs important for transcriptional factor binding. During *in vitro* growth, the M. smegmatis/pJK mutants selected showed a stable level of fluorescence. The P_Hsp60_ promoter placed in the same plasmid backbone as the pJK series induced a fluorescence intensity comparable to that obtained with the weakest P_L5_ promoter, thus indicating the high strength of P_L5_. Heat shock treatment did not increase the fluorescence of M. smegmatis/pJK or pJHsp60 (not shown). It could be argued that fluorescence may not serve as a good parameter to determine protein expression and ultimately promoter activity. However, here we have demonstrated that fluorescence determined by flow cytometry correlates with the total amount of GFP expression in SDS-PAGE even though only six promoters were used.

In this study, we observed mutations throughout the whole sequence, including the −10 and −35 regions of the promoters, exhibiting high, medium, or low strength. Interestingly, all of the midstrength promoters (pJK-B7, pJK-E6, and pJK-D6) showed a mutation at the first position of the −10 region (C → T). Also, the weakest promoter that we identified, in pJK-C1, is the only one with a mutation at the predicted transcriptional start site (A → G).

Heterologous expression in recombinant bacteria can be deleterious or lethal. Given the relatively low mycobacterial growth rate, where faster-growing strains like M. smegmatis and M. fortuitum have a doubling time of 2 to 3 h and M. bovis BCG and M. tuberculosis have a doubling time of 20 to 24 h (33), eGFP synthesis undoubtedly represents a metabolic burden and stress through consumption of ATP, nucleotides, and amino acids and usage of ribosomes and tRNAs that may displace the expression of endogenous proteins and hence inhibit the bacillus's growth. GFP itself can also be toxic to the host (26). Nevertheless, in this study, the only strain significantly different from the wild type in growth was M. smegmatis/pJK-C1. This strain appears to have a lag at early time points but reaches a similar OD at later time points. However, it is unlikely that low and not high eGFP expression could inhibit growth, as observed by others (34).

To evaluate the utility of P_L5_ promoters for driving foreign antigen expression in BCG, the *sm29* gene from S. mansoni was cloned in place of the *egfp* gene downstream of a strong (pJK-F8), an intermediate (pJK-B7), or a weak (pJK-C1) mutant promoter. rBCG expressed Sm29 at different levels, which corresponded to their eGFP-measured promoter strength. pJK-F8 was not evaluated since no colonies of rBCG were obtained, even after several attempts.

rBCG/pJK-B7 clones were 9-fold more fluorescent than rBCG/pJK-C1 clones (average FITC values of 27,300 and 3,200, respectively). To achieve such a difference, Bourn et al. used an enrichment method to select for high-copy-number variants of plasmid pORI101 with GFP expression 7-fold higher than that of the original (16). Although this strategy can be useful in obtaining significant expression of cloned genes, some disadvantages, such as a metabolic burden and plasmid instability, may reduce the usefulness of the system.

Since pJK plasmids are extrachromosomal, their copy number could be partially responsible for the differences in fluorescence or Sm29 expression observed among mycobacterial hosts. The origin of replication of pJH-*egfp* and pJK is derived from pAL5000 of M. fortuitum, which is widely used in mycobacterial shuttle plasmids such as pMV261, pLA71, and pMIP12. Its copy number in M. smegmatis was determined as 3 by Southern blot assay (35), 8 by antibiotic resistance (36), and 23 by quantitative PCR (37). The variation of the copy number among these studies can be explained by the different methods of quantification used but also by the differences in plasmid structure. Despite the same pAL5000-derived origin of replication, other features such as the resistance marker, size, and some other elements are also different. In our study, all of the features are the same, with the exception of a few nucleotides at the promoter sequence. Thus, we expect to minimize the effect of the plasmid copy number on differential expression. Besides that, although there are minor variations in fluorescence during the growth of recombinant M. smegmatis, the fluorescence values do not intersect each other.

In conclusion, this study demonstrates the utility of the rational design of systems to obtain a range of expression of target proteins in mycobacteria. The systems described present an alternative way to obtain the expression of foreign genes in mycobacteria and to design a new generation of rBCG vaccines that optimize the host immune response for protection.

## Supplementary Material

Supplemental material
