# Supplementary material for: New Recombinant Mycobacterium bovis BCG Expression Vectors: Improving Genetic Control over Mycobacterial Promoters
Source: Appl Environ Microbiol. 2016 Apr 4;82(8):2240–6. doi: 10.1128/AEM.03677-15 (PMC4959472; doi:10.1128/AEM.03677-15)
Supplement: Supplemental material [file supp_82_8_2240__index.html]

New Recombinant Mycobacterium bovis BCG Expression Vectors: Improving Genetic Control over Mycobacterial Promoters — Supplemental material 

# New Recombinant Mycobacterium bovis BCG Expression Vectors: Improving Genetic Control over Mycobacterial Promoters

## Supplemental material

- Supplemental file 1 -

  Growth curves for *M. smegmatis*::pJK (Fig. S1); SDS-PAGE of recombinant *M. smegmatis* harboring pJK plasmid series (Fig. S2).

  PDF, 127K
