# Supplementary material for: New Recombinant Mycobacterium bovis BCG Expression Vectors: Improving Genetic Control over Mycobacterial Promoters
Source: Appl Environ Microbiol. 2016 Apr 4;82(8):2240–6. doi: 10.1128/AEM.03677-15 (PMC4959472; doi:10.1128/AEM.03677-15)
Supplement: Supplemental material [file AEM.03677-15_zam999117056so1.pdf]

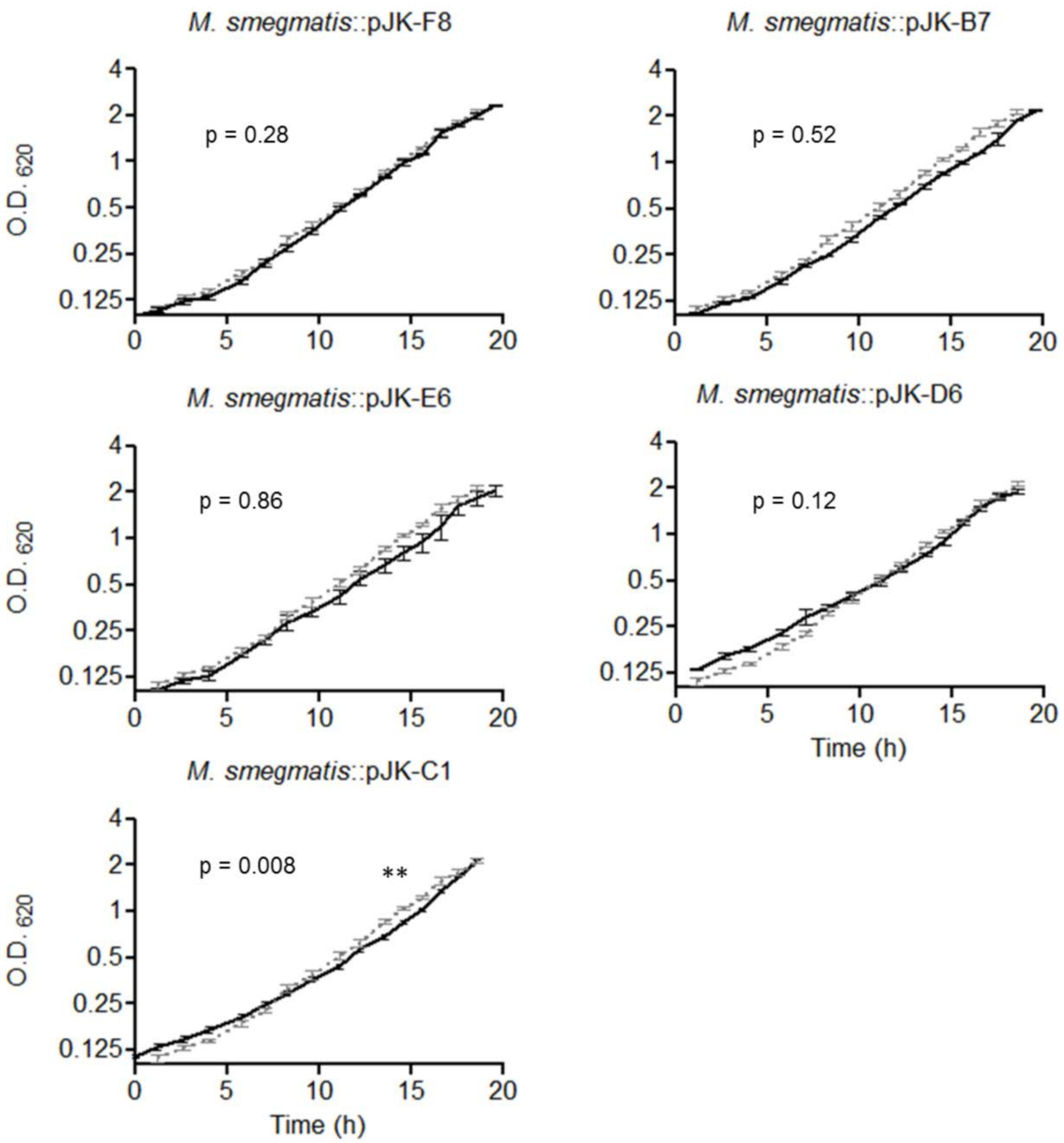

Supplementary Figure 1: Growth curves of *M. smegmatis*::pJK.*M. smegmatis*::pJK were cultured in MB7H9 broth and turbidity measurements made at time points up to 20 hs. The growth curve of the wild type strain is also indicated (gray line). All strains but pJK-C1 demonstrate a growth curve similar to the wild-type strain. The difference in growth was assessed by using non-linear regression and *p* values are indicated in each graph.

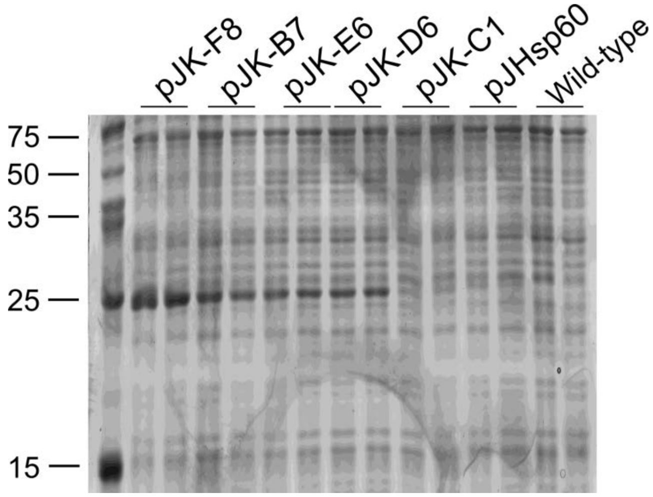

Supplementary Figure 2: SDS-PAGE of recombinant *M. smegmatis* harboring pJK plasmid series. Soluble protein extracts of late-log phase cultures of *M. smegmatis* transformed with pJK plasmids or pJHsp60 (10 µg) were subjected to SDS-PAGE and Coomassie staining. The GFP predicted MW ~28 kDa. Molecular weight markers standard are indicated on the left.
